# A risk‐based approach to identifying oligometastatic disease on imaging

**DOI:** 10.1002/ijc.31793

**Published:** 2018-10-09

**Authors:** Nandita M deSouza, Clare M Tempany

**Affiliations:** ^1^ Cancer Research UK Imaging Centre at The Institute of Cancer Research and The Royal Marsden NHS Foundation Trust Sutton United Kingdom; ^2^ Department of Radiology Brigham & Women's Hospital, Harvard Medical School Boston MA

**Keywords:** oligometastases, imaging, morphology, metabolic, phenotype

## Abstract

Recognition of <3 metastases in <2 organs, particularly in cancers with a known predisposition to oligometastatic disease (OMD) (colorectal, prostate, renal, sarcoma and lung), offers the opportunity to focally treat the lesions identified and confers a survival advantage. The reliability with which OMD is identified depends on the sensitivity of the imaging technique used for detection and may be predicted from phenotypic and genetic factors of the primary tumour, which determine metastatic risk. Whole‐body or organ‐specific imaging to identify oligometastases requires optimization to achieve maximal sensitivity. Metastatic lesions at multiple locations may require a variety of imaging modalities for best visualisation because the optimal image contrast is determined by tumour biology. Newer imaging techniques used for this purpose require validation. Additionally, rationalisation of imaging strategies is needed, particularly with regard to timing of imaging and follow‐up studies. This article reviews the current evidence for the use of imaging for recognising OMD and proposes a risk‐based roadmap for identifying patients with true OMD, or at risk of metastatic disease likely to be OM.

## Introduction

The development of metastases is driven by a variety of tumour and host characteristics^1^, ^2^ such as escape of tumour cells from the primary site, their survival in the circulation and lymphatics, seeding and invasion at a host metastatic site, angiogenesis and immune escape.^3^, ^4^ Where metastases are few in number (<3 lesions in <2 organs), the term Oligometastatic disease (OMD) has been coined.^5^ This lies between the situation of organ‐confined localised disease and widespread (poly‐) metastases (PM) and carries a different prognostic implication.^6^, ^7^, ^8^ Patients with OM may potentially be treated with curative intent or attempt at long term disease or symptom control, 9, 10, 11 which means that their management often may differ from those with PM disease. Therefore, the identification of the true OM state on imaging is crucially important. Particular tumour types are predisposed to an OM state; these include colorectal, prostate, renal, sarcoma and lung cancer. It is possible to potentially predict the probability of a likely oligometastatic state from baseline tumour evaluation that documents the primary tumour features on imaging (size, volume, metabolism), histological typing and/or genetic profiling as they reflect tumour burden and metastatic propensity.^12^, ^13^, ^14^, ^15^

The conditions that favour development of OM as opposed to a PM have been modelled by Withers and Lee.^16^ They recognise 4 predisposing factors. Firstly, a long interval between surgical removal of the primary and the appearance of a single metastasis – late appearance of a metastasis implies a long doubling time of a lesion that was undetectable at the time of surgery and therefore either solitary lesion or with a limited number of followers. Secondly, a single slow‐growing metastatic deposit with a large difference in volume between it and any measurable followers indicates a greater probability of OMD. Thirdly, a solitary metastasis in the presence of a large primary tumour is also more likely to indicate an OM state and fourthly effective chemotherapy that wipes out micrometastatic burden is more likely to predispose to an OM recurrence.

This article reviews the role of imaging in defining OMD. It acknowledges the limitations of current imaging modalities, which may be poorly sensitive, and the challenges of implementing newer, more sensitive imaging modalities that largely remain nonstandardised and invalidated, issues that have been recently addressed comprehensively.^17^ Here we discuss the optimal timing and frequency of follow‐up with the relevant imaging techniques in relation to tumour biology and specifically include evaluation of features of the primary tumour that may be used to assess metastatic risk.

## Metastatic Risk Assessment

### Histological and genetic features of the primary tumour: can OM and PM disease be predicted?

It has long been known that histological type and grade of a tumour is indicative of metastatic risk.^18^, ^19^ A breast cancer series of >2,000 patients from 8 German centres showed that metastases at presentation were more commonly associated with grade 3 lobular histology and a Luminal B phenotype (HER2 positive).^20^ In primary soft‐tissue sarcoma, where histological type varies widely, tumour grade has been shown to be independent of histological subtype for predicting metastatic relapse.^21^ In renal cell cancer, the Leibovich score (clear cell subtype) and UICC/AJCC grading (other subtypes) are used for risk stratification: high risk patients have a ~60% risk of recurrence at 5 years versus 10% in low risk disease. Often recurrences are solitary or oligometastatic (41% of 68 patients in one retrospective study).^22^ However, although histological grading identifies metastatic risk, there is no data to indicate a preferential distinction between OM or PM phenotypes.

Comparison of the genetic features of patients with OM who subsequently turn out to be PM vs. those who were truly OM has revealed interesting differences. Distinct microRNA expression patterns were found in a small study of 34 patients, all of whom had received radiotherapy with curative intent to their oligometastases, these patterns were not just evident in the oligometastasis, but also in the primary tumour itself.^23^ It was possible in this pilot study to prioritise these microRNA's that differed between primary tumours known to develop OM versus PM and use them to predict the OM state in metastatic samples. The pathways targeted by these microRNA's relate to suppression of cellular adhesion, invasion and motility. In particular, four microRNAs encoded in the 14q32 locus (miR‐127‐5p, miR‐369‐3p, miR‐544a, and miR‐655‐3p) were associated with an OM phenotype in clinical metastasis samples. I*n vitro* assays of adhesion and invasion using metastatic cell lines transfected with these microRNA's resulted in a significant decrease in adhesion to Matrigel as compared to nontargeted control transfected cells. Further, ectopic expression of selected 14q32‐encoded miRs or stable repression of targeted genes by shRNAs led to reversal of a PM phenotype to an OM phenotype.^24^

### Imaging the primary tumour to distinguish OM from PM

In addition to T‐staging, the phenotype of a tumour on imaging is a well‐established predictor of disease progression/outcome. In rectal cancer, pre‐operative high resolution T2‐W MRI can distinguish prognostic groups based on assessment of depth of extramural spread, relationship of the tumour edge to the mesorectal fascia and extramural venous invasion.^25^ Likewise, in synovial sarcoma, T1 stage, as well as heterogeneous enhancement, interfascial extension and perilesional oedema indicate a higher incidence of metastatic disease.^26^ Angiogenic capability of tumours is another imaging feature associated with metastatic potential: inhibiting VEGF in subcutaneous breast cancer models substantially reduced the development of metastases.^27^ High expression of metastasis associated protein (MTA1) in oral squamous cell carcinoma has been associated with increased tumour angiogenesis and progression to metastasis,^28^ while in papillary thyroid cancer, angiogenesis as measured by immunohistochemistry of microvessel density is more intense among metastatic tumours.^29^ However, there are as yet no data indicating a differential angiogenic capability of an OM versus a PM phenotype.

Evidence also links functional imaging‐based tumour properties to metastatic disease, prognosis and survival. In non‐small cell lung cancer (NSCLC), quantitative measures of PET tracer uptake – (so called‐Maximum Standardised Uptake Value (SUV_max_)) has been shown to be able to predict occult nodal metastases from the metabolic activity of the primary tumour.^30^ In a multivariate analysis SUV_max_ was independent of tumour size and importantly type of tumour, for predicting the presence of occult lymph node metastasis.^31^ In an initial study of 63 patients, an Optimal cut‐off value of 8.8 SUV_max_ of the primary tumour was shown to predict occult metastases in NSCLC^32^ although in another larger cohort (*n* = 163), an SUV_max_ of 7 was deemed to be the best threshold for indicating metastatic risk; a value <7 was shown to be an independent prognostic factor for metastasis‐free survival.^33^ High SUV as an independent biomarker of prognosis has been borne out in lung cancer (meta‐analysis of 21 lung cancer studies had a combined hazard ratio of 2.08),^34^ in breast cancer (hazard ratio 2.39)^35^ and in soft‐tissue sarcoma (hazard ratio 3.75),^36^ where it has been related to mitotic count.^37^ In renal cell cancer, the apparent diffusion coefficient (ADC)^38^ derived from diffusion‐weighted MRI and SUV_max_ from ^18^FDG‐PET have been shown to be statistically significant independent risk factors for high histological grade and hence of metastatic risk.^39^

Heterogeneity of morphological and functional imaging features is proving of interest in predicting metastatic risk. Although the data from ^18^FDG PET remains controversial^40^, ^41^ entropy measures from histogram analyses of MRI based tumour ADC can predict positive nodal status in rectal cancers^42^ and in gastric cancers, while in soft‐tissue sarcomas, first order statistics from ADC, which relate to signal variability and to entropy and dissimilarity, were higher in high grade tumours.^43^

Finally, the initial response of the primary tumour to a given chemotherapy is also a powerful prognostic indicator^44^, ^45^, ^46^, ^47^ for subsequent development of metastases. Data from the International Metastatic Renal Cell Carcinoma (mRCC) Database Consortium showed that solitary versus multiple metastases were commoner in long‐term survivors (ratio 0.3 vs. 0.2) treated with targeted agents, mainly VEGF and mTOR inhibitors.^48^ In soft‐tissue sarcomas (*n* = 34); freedom from distant metastasis was superior if treatment‐induced tumour necrosis was 90% or greater (84.6% vs. 19.9%, *p* = 0.02) indicating likely control of micrometastases in these patients.^49^ In metastatic colorectal cancer, the hazard ratio for progression‐free survival among patients with wild‐type‐KRAS tumours was 0.68 (95% CI, 0.50 to 0.94) in favour of a cetuximab‐FOLFIRI combination treatment compared to a FOLFIRI alone group (*n* = 599 in each arm)^50^ emphasising the critical role of effective chemotherapy in controlling distant disease. In 228 rectal cancer patients followed‐up for a median of 49 months, pathological response as defined by tumour regression grade was the only independent factor for predicting subsequent metastases,^51^ further highlighting the vital role of effective chemotherapy in determining metastatic risk. As chemotherapy becomes more effective, it is expected that the likelihood of OM as opposed to PM disease will increase.

## Imaging for detecting OMD

### Available modalities

The commonest sites of metastases (OM or PM) are liver, lung, skeletal and nodal. While the first two require imaging with dedicated organ coverage, imaging requirements for the latter two necessitate whole‐body techniques. Important considerations are the selection of the imaging technique that delivers maximal sensitivity and specificity for disease detection, appropriate organ coverage and the optimal timing and intensity of imaging follow‐up.

CT scans detect metastases within soft‐tissues and bone based on a change in tissue morphology and density. MRI can also detect functional features such as tumour vascularity, cellularity, stiffness and metabolism. Bone scintigraphy^52^, ^53^, ^54^ reflects bone remodelling and is not specific for metastatic tumour itself, with sensitivity around 85% for the identification of bone metastases on a patient level with a specificity of 75–80%. Glucose avidity on ^18^FDG‐PET is directly related to metabolic activity and turnover of tumour cells and is particularly effective in recognising OM or monometastatic disease.^55^ Despite the sensitivity of, ^18^FDG PET‐CT for metastasis detection in lung cancer, it is not routinely recommended for follow‐up^56^ although it is common practice in academic centres.

If metastatic risk is more likely at a particular site, high‐resolution imaging should focus on the region‐of ‐interest (e.g. CT or MRI with liver‐specific contrast agents in colorectal disease, or high‐resolution chest CT in sarcoma). Conversely, full body coverage with MRI or PET needs to be retained where skeletal or multiple organ involvement is more likely (e.g. for prostate cancer). In addition to spatial resolution, tumour to background contrast is vital for optimal sensitivity of disease detection. A meta‐analysis of nine diagnostic accuracy studies (537 patients with 1,216 lesions) and four change‐in‐management studies (488 patients with 281 lesions) emphasises the vital importance of tumour to background contrast in lesion detection. The per‐lesion sensitivity and specificity for contrast‐enhanced‐MRI ranged from 86.9–100.0% and 80.2–98.0%, respectively, compared to 51.8–84.6% and 77.2–98.0% for contrast‐enhanced‐CT because of the superior image contrast of the former.^57^

Sensitivity and specificity of PET imaging for detecting OM may be optimised further by disease‐specific radiotracers directed against tumour specific antigens. A pitfall of these techniques lies in the differential expression of the antigens in tumour versus normal tissue and in changing tumour biology. For example, 18F‐fluorodihydrotestorsterone (^18^F‐FDHT) a radio‐labelled dihydrotestosterone analogue, directly targets the androgen receptor (AR) on tumour cells. However, testosterone levels above castration level means that competitive binding can hamper accurate FDHT evaluation. Similarly, ^68^Ga‐labelled prostate‐specific membrane antigen (PSMA) used to image prostate cancer may be falsely negative in the liver where a high background activity can potentially obscure lesions, or when liver metastases tend to lose PSMA‐expression in advanced metastatic disease. Sensitivity, specificity and accuracy against a surgical gold standard for lymph node detection in a multicentre study for ^68^Ga‐PSMA PET in the primary setting were 53%, 86% and 76% but improved with higher surgical sampling, increasing to 67%, 88% and 81% in a subgroup with of patients with ≥15 lymph nodes removed.^58^ Imaging data for these newer agents has been validated and shown to reflect target expression: a small but intensive study of targeted agents such as ^18^F‐DHT and ^18^F‐ES (Estradiol) showed that semi‐quantitative androgen or oestrogen receptor expression on immunohistochemistry and ^18^F‐DHT or ^18^F‐ES uptake respectively on PET was correlated. Moreover, using optimal cut‐offs (SUV_max_ of 1.94 for ^18^FDHT‐PET and 1.54 for ^18^FES‐PET) sensitivities of 91% and 100% and specificities of 100% and 100% respectively were achieved.^59^

**Figure 1:**
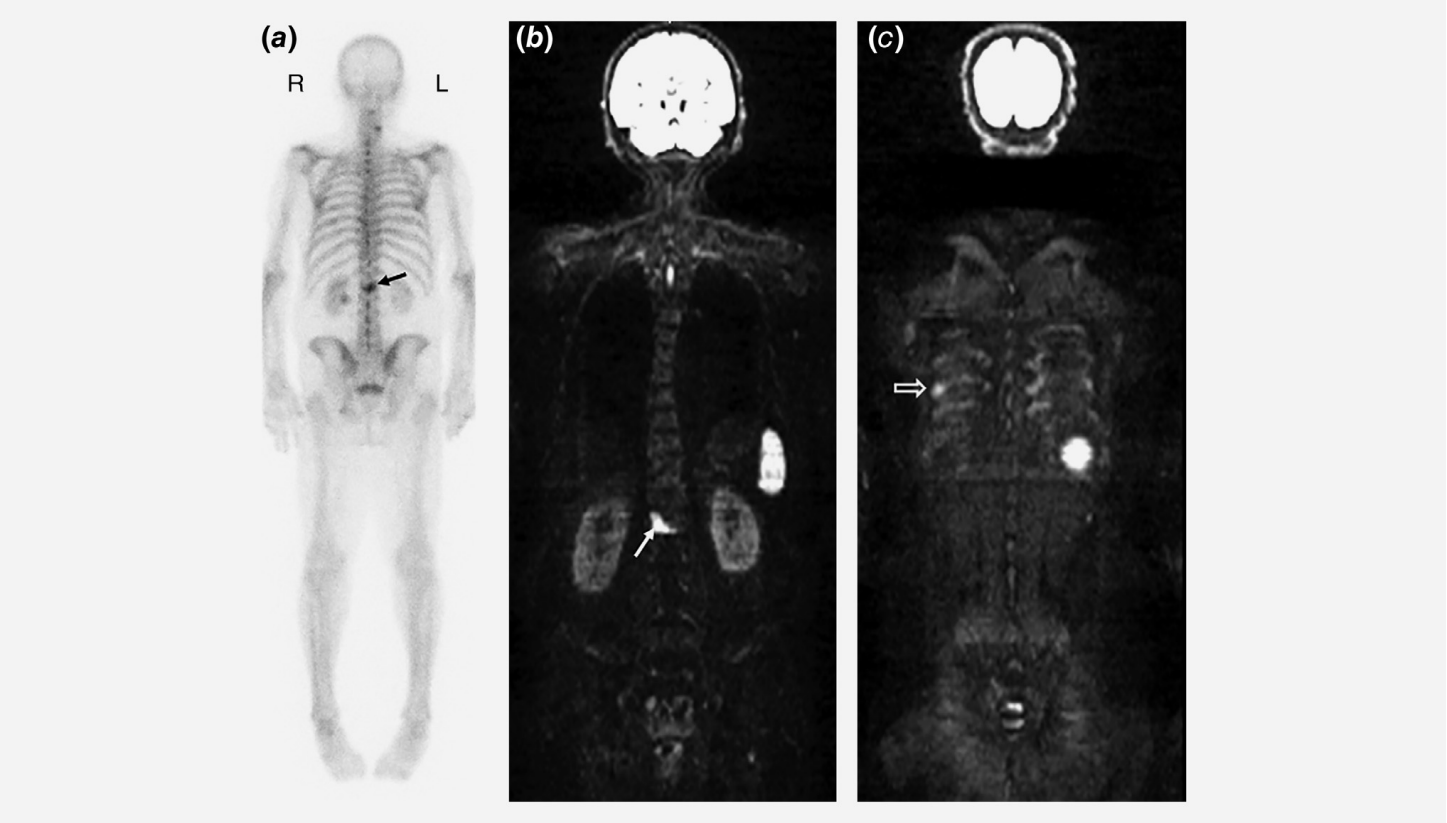
Eighty‐one years. *old male with oligometastatic prostate cancer:* The patient presented 13 years previously with T3a, Gleason 4+4 prostate disease. He was treated with radiotherapy complemented by androgen blockade for 4 years. He experienced biochemical recurrence 10 years post diagnosis. Bone scintigraphy (*a*) showed a solitary metastasis in the lumbar spine (arrow), whole body MRI (*b* = 900 mm^2^/s) confirmed this lesion (*b*, arrow) and revealed the additional rib lesion (*c*, open arrow), indicating the value of utilizing the most accurate imaging modality at the outset prior to planning management.

Whole‐body MRI has the advantage of combining morphological data (T1 and T2‐W imaging) with functional information (diffusion‐weighted and dynamic contrast‐enhanced imaging) thus simultaneously exploiting multiple contrast mechanisms to image and detect the presence of disease. As diffusion‐weighted and contrast‐enhanced MRI data are fundamentally quantitative, thresholding can be set so that the images display the required sensitivity and specificity. Anatomic images are most useful at the early stages, i.e. at presentation or first biochemical recurrence. At later stages of disease, morphologic images can be more difficult to interpret, especially with the appearance of new lesions or reactivation of previously responding metastases on a background of treated lesions. Here the role of diffusion‐weighted sequences becomes crucial.^60^ Inverted greyscale images of maximum intensity projections of high b‐value images in conjunction with the morphological images are helpful to detect metastatic foci (Fig. 1).^61^ Qualitative assessment of inverse maximum intensity projection of high b‐value images 62 visually appear like radioisotope studies and need comparison with morphological imaging for verification. Although highly sclerotic metastases may be missed on high b‐value DWI images, they are correctly identified on anatomic MR images, which also identifies any T2 shine through effect. Inter‐observer agreement for reading of WB‐MRI images including DWI has been evaluated as very good (*K* = 0.87 [Confidence Interval 0.66; 1.00]) in several studies^62^, ^63^ outperforming a moderate inter‐observer agreement for bone scintigraphy (*K* = 0.60 [Confidence Interval 0.26; 0.78])^64^ ADC maps provide information on the cellularity, viability and changes over time and are essential for lesion follow‐up under treatment. Finally, applying an ADC threshold to the images enables automated measurements of the global volume of metastatic disease, which can be exploited as prognostic and response biomarkers.^65^, ^66^, ^67^

**Table 1:** Features of the primary tumour that aid distinguishing oligo‐ from poly‐metastatic disease

	Oligometastatic	Polymetastatic
Histological	Tumour grade in many cancer subtypes (breast, renal, sarcoma) indicates metastatic risk^18^, ^19^, ^20^, ^21^, ^22^ but has not been shown to predict OM vs. PM	
Genetic	microRNA expression linked to OM phenotype^23^	Genetic heterogeneity of the primary tumour is a risk for metastatic disease in general
Morphology (Size, shape)	Large, slow growing primary tumour^16^ Metastases small and uniform with nonspecific shape or imaging features.	No distinguishing size or shape features‐but typically multi‐focal and heterogeneous in size and shape
Functional imaging features	Angiogenic features, maximum standardised uptake value on FDG‐PET, apparent diffusion coefficient on MRI are all linked to tumour grade and metastatic risk,^25^, ^26^, ^27^, ^28^, ^29^, ^30^, ^31^, ^32^, ^33^, ^34^, ^35^, ^36^, ^37^, ^38^, ^39^ but no evidence for their use in distinguishing OM from PM	
Response to chemotherapy	Good initial response to chemotherapy and high tumour regression grade, indicate good control of micrometastases and favour OM at recurrence^16^	Poor or very mixed initial response to chemotherapy favours PM at recurrence

### Challenges in implementation

Despite the exponential availability of imaging and the declining costs of genetic analyses needed to characterise tumours, several challenges in effective recognition of OM remain. Imaging detection sensitivity depends on the modalities, spatial and contrast resolution. Spatial resolution depends on hardware and software capabilities of the imaging equipment (transducer frequency, detector arrays, magnetic field strength and gradients applied). With MRI, spatial resolution is a trade‐off against coverage, so can be as high as 0.5mm^3^ for dedicated organ imaging but is around 50mm^3^ for whole body techniques. Generally, lesions <5 mm are considered undetectable by RECIST (Response Evaluation Criteria in Solid Tumours) criteria, as specificity is low for small volume lesions. A 5 mm lesion effectively has a volume of 65mm^3^ and therefore approximately 150–350 million cells (based on a cell size of 200–400 microns,^68^ making this the lower limit of detection of lesions with current standard imaging techniques. The spatial resolution of PET imaging techniques is dependent on the energy of the tracer and the sensitivity of the detector arrays. For ^18^F, this is of the order of 5‐7 mm which means that generally lesions of <1 cm are not reliably detected. Contrast resolution on the other hand, depends not only on the imaging technique but may vary with tumour biology. It is vitally dependent on differences in properties between the tumour and the background tissue (e.g. density for CT, tissue water relaxation after RF excitation in a magnetic field in MR, uptake of extrinsic radiotracers on PET). Differences in cellular density and extracellular matrix composition of metastases at different anatomical locations may affect image contrast and profoundly influence detection sensitivity. Poor image contrast may mean that even a high spatial resolution technique such as CT (30 microns) does not deliver the desired detection sensitivity.

Radiotracer techniques that are target‐specific are highly sensitive (e.g. targeting somatostatin receptors has been widely used for imaging neuroendocrine tumours)^69^ but may suffer from specificity issues if the target is expressed more generally (HER2).^70^ Of note, ^68^Ga‐PSMA PET can be false negative in up to 5% of patients with prostate cancer, due to absent or low expression of PSMA on prostate cancer cells. It also has been reported that in advanced metastatic castration‐resistant prostate cancer, metastases (mainly in the liver) can lose PSMA‐expression.^71^, ^72^, ^73^

Accurate detection of metastatic lymph nodes remains the holy grail of oncological imaging. Identification of abnormally enlarged lymph nodes is the domain of CT where the sensitivity of detection of enlarged nodes depends upon the size threshold utilised. In RECIST 1.1, nodes with short axis ≥ 10 mm but <15 mm are considered pathological, although nontarget lesions. CT is unable to detect architectural changes within normal‐sized (<10 mm) lymph nodes, which results in a low sensitivity (40%) as the majority of metastases are microscopic. The average specificity however is around 80% against a surgical gold standard because reactive or inflammatory change within lymph nodes results in false positives. Despite the high spatial resolution of CT, the poor contrast resolution means that it performs less well than MRI.^64^ ^18^F‐Choline PET also has been trialled in several studies for metastatic node detection: in 130 prostate cancer patients at high‐risk for extra‐capsular disease who underwent radical prostatectomy, with 912 lymph nodes sampled ^18^F‐Choline PET/CT showed a better performance than CT for detecting nodal involvement, particularly for metastases greater or equal than 5 mm in size (sensitivity 66%, specificity 96%).^74^ However, this low sensitivity means that it does not merit routine use. RECIST 1.1 criteria^75^ on either CT or MRI therefore remains the mainstay for identifying nodal metastases.

Validation of OMD on imaging remains hugely problematic. It is not feasible to biopsy multiple sites, particularly in bone and brain. Confirmation of accuracy of new imaging agents is highly dependent on what is known from preclinical studies, or on longitudinal follow‐up observational data. The imaging community have produced an imaging biomarker roadmap that addresses these issues, which is being widely implemented.^76^

## Timing and Mode of Imaging follow‐up

**Figure 2:**
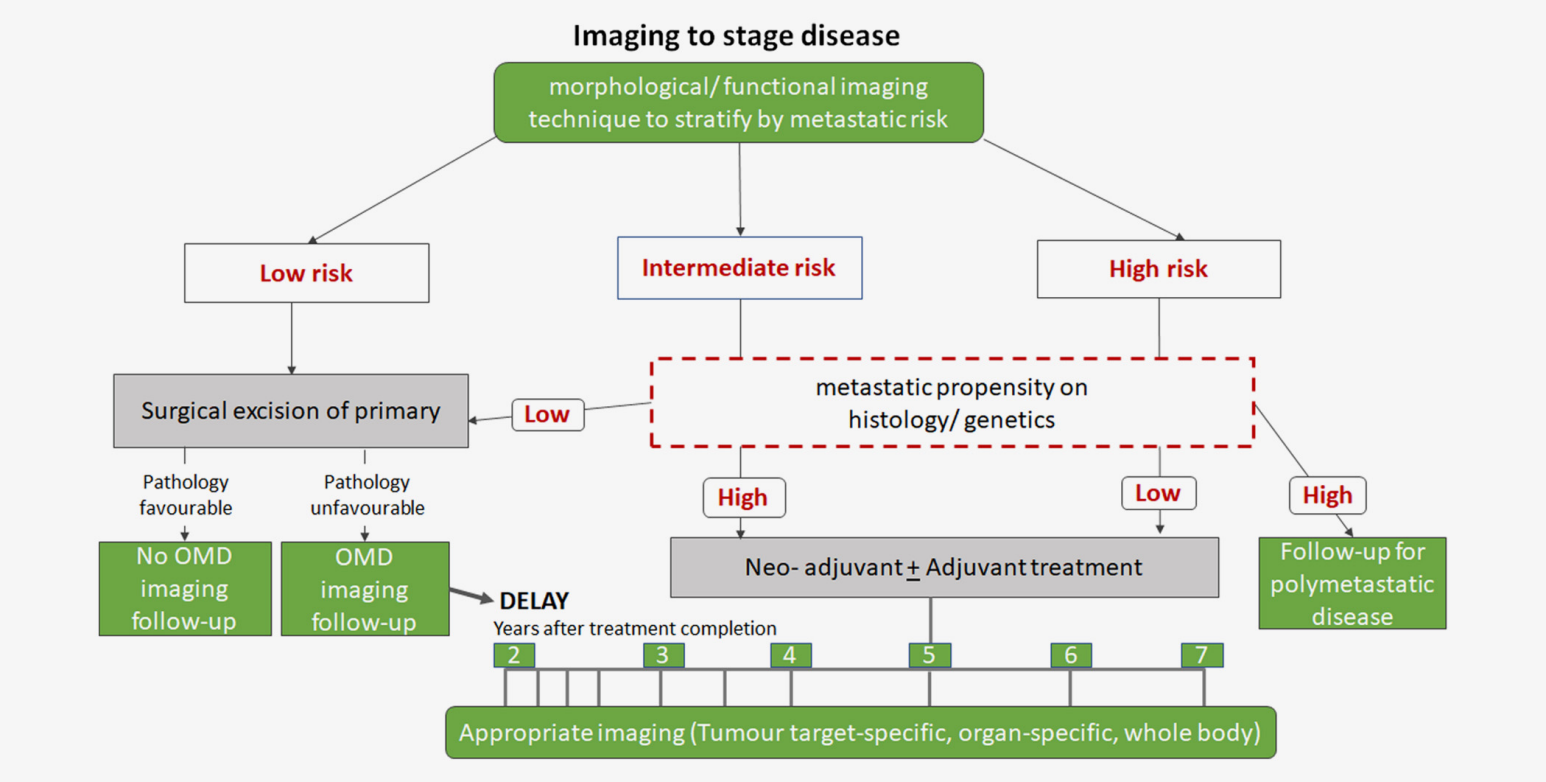
Proposal for an imaging workflow for detection of oligometastatic disease.[Color figure can be viewed at wileyonlinelibrary.com]

The timing of longitudinal or follow‐up imaging studies is an important consideration. Conventionally, after the effective treatment of the primary tumour, patients are often imaged 3‐monthly for the first year, 6‐monthly for the next 2 years and then annually or as the clinical situation demands. Although the rationale for this is geared to detecting recurrence at the primary site it does not in fact fulfil the need for detecting OM where the likelihood of detecting disease in the first year is low with increasing likelihood thereafter. True OM amenable to curative treatment will remain undetectable until they reach a 2^30^ cell burden and are unlikely to be manifest in the first year. Over a decade ago Singh et al. followed 369 patients with Stage T1‐T3aN0‐NXM0 prostate cancer for 10‐years who were treated with external beam radiation with curative intent to a mean dose of 65Gy. There was a better overall survival in patients with <5 metastases than those with more numerous lesions.^77^ The location of these metastases was largely in the spine and pelvis and only 2 patients had disease elsewhere (lung, liver, brain). Moreover, the mean interval from the date of the initial diagnosis of prostate cancer to the time of diagnosis of bone metastases was 4.9 years (range 0.7–10.5) in the group with <5 lesions, compared with 3.3 years (range 0.5–10.7) in the group with >5 lesions (*p* = 0.02). Dominant patterns of metastasis to suggest a multi‐step hierarchical order of metastasis did not occur. Therefore, a follow‐up protocol for detection of OM in prostate cancer would ideally be intense and increased around the 4 year mark and employ the most sensitive techniques that covered the spine and pelvis, either with a targeted radioisotope or whole‐body scanning, increasingly done with MRI (Fig. 2). In nonmetastatic NSCLC, systematic interrogation of patients treated with definitive radiation (≥60Gy) showed that there were different patterns of metastatic spread.^78^ This means that a whole‐body, rather than a sequential targeted imaging approach for detection is warranted.

**Table 2:** Advantages and Limitations of commonly available imaging modalities for detecting and assessing metastatic disease and treatment response

	Advantages	Limitations
Bone Scintigraphy	Widely available Low cost Easy to implement, especially if high suspicion of bone metastases	Poor sensitivity (50%) Radiation dose (~3‐4 mSv) Response assessment is challenging
CT	Widely available Relatively low cost Soft tissue and bone delineation High contrast in lung	Sensitivity limited by poor soft tissue contrast within abdomen and pelvis (liver, peritoneum) Radiation dose (~10‐14 mSv) Relative lack of standardisation Requires Iodinated contrast material with potential nephrotoxicity
Organ‐specific MRI	High inherent soft tissue contrast, especially with diffusion‐weighted imaging No ionising radiation Availability of organ‐specific contrast agents for liver imaging	Poor sensitivity in lung due to susceptibility effects and motion artefact Relative lack of standardisation High cost May require Gadolinium chelates with associated potential toxicities
Whole‐body MRI	Whole‐body coverage Multiple tissue contrasts yield high sensitivity No ionising radiation	Specificity low Lack of validation studies Limited availability High cost, Long examination time (45 mins) May be poorly tolerated by patient
^18^FDG‐PET	High sensitivity^55^ Whole‐body coverage Combined metabolic (FDG) and morphological (CT) data	Radiation dose (~14 mSv) High cost Limited availability Limited spatial resolution for subcentimeter lesions
Receptor specific radiotracers (18F‐DHT, 68GA‐PSMA)	High specificity^58^ Whole body coverage Combined tumour receptor specific and morphological (CT) data	High cost Availability limited to tertiary cancer centres Radiation dose Follow‐up where receptor expression is negative yields false negatives

## Summary

Recognition of the metastatic potential of a tumour is crucial in determining patients’ management pathway. Metastatic propensity is traditionally based on histologic types although increasingly genetic profiling is being used to stratify patients to various management options with surgery, radiotherapy and chemotherapy/Immunotherapy as appropriate. The addition of an imaging phenotype into this paradigm can have a major impact on treatment decisions. Thereafter, a tumour target‐specific protocol that is organ‐specific or requires a whole‐body approach can be decided on, depending on the tumour type and likely mode of spread. A regime of more frequent follow‐up between 2 and 4 years after completion of treatment rather than early post‐treatment is most likely to favour earlier recognition of OMD. Achieving an appropriate cost‐effective surveillance program for imaging patients at risk of OMD opens the door to new therapeutic strategies for these patients with the potential for cure.
